# Leveraging the Client-Provider Interaction to Address Contraceptive Discontinuation: A Scoping Review of the Evidence That Links Them

**DOI:** 10.9745/GHSP-D-21-00235

**Published:** 2021-12-31

**Authors:** Kendal Danna, Alexandra Angel, Jamee Kuznicki, Laetitia Lemoine, Klaira Lerma, Amanda Kalamar

**Affiliations:** a Population Services International, Washington, DC, USA.; b Stanford University, Stanford, CA, USA.; c University of Texas at Austin, Austin, TX, USA.

## Abstract

Despite considerable investment and effort, unmet need for contraception remains an obstacle to improved family planning outcomes. One influencing factor is the frequency of contraceptive discontinuation among users who desire to prevent pregnancy, often due to method-related concerns and side effects. Contraceptive users have the right to be supported during counseling to voluntarily choose methods that align with their individual needs and preferences. Contraceptive counseling, as a key component of quality of care, is particularly important for providers to reduce unmet need among their clients. This scoping review examined the state of the evidence on contraceptive counseling and its impact on discontinuation. The review first examines the association between quality of care and contraceptive discontinuation, then looks to what the current body of evidence suggests are women's contraceptive counseling priorities, and lastly, explores whether specific counseling tools and approaches have been evaluated with discontinuation as an outcome. The results identified general principles and priorities for good counseling including person-centeredness, client-tailored information exchange, clear and concise information on side effects and bleeding changes, reducing providers' implicit and explicit biases, and trust and respect between the client and provider. The review of the literature also found that evidence to support the use of specific counseling tools and approaches to reduce contraceptive discontinuation is insufficient; research should be designed to determine which specific elements of the client-provider interaction can be improved to significantly impact contraceptive discontinuation. This evidence could inform how the global community of practice might improve and leverage specific counseling approaches and tools to address the most common predictors of discontinuation.

## Abstract (precis)

After examining existing evidence on contraceptive counseling and its impact on discontinuation, this scoping review identifies principles and priorities for better rights-based counseling, yet also illuminates the need for more evidence to understand relationships between counseling and discontinuation.

## Teaser

**Key Findings:**

- Reasons for discontinuation, while complex, include contraceptive counseling as an element that could be optimized to better support clients to choose methods that will enable voluntary continuation.
- Information exchange during counseling—in particular how providers do or do not prepare clients for side effects—is associated with contraceptive discontinuation.
- There is a need for evidence-based understanding of associations between common counseling approaches/tools and discontinuation. This lack of evidence incurs risk of using techniques that do not effectively lead to client satisfaction.

**Key Implications:**

- Policy makers should lead a call for better understanding of associations between existing and new counseling approaches and their impact on health outcomes, especially contraceptive discontinuation while in need.
- Program managers should consider using more evidence-based counseling approaches as part of a strategy for decreasing method-related discontinuation of contraception. Where evidence does not support the impact of existing approaches on health outcomes, improved tools and strategies should be developed and evaluated before use.
- Implementers should include indicators of interpersonal relations and information exchange as key measures of person-centered contraceptive counseling.

## INTRODUCTION

International family planning stakeholders have been working to address unmet need for decades, but the problem remains a widespread, complex challenge. Addressing contraceptive discontinuation, a compounding factor, could reduce unintended pregnancies that result from unmet need.

Discontinuation is not inherently problematic; contraceptive users should have the ability and agency to discontinue use at any time and for any reason. However, an estimated 1 in 5 mistimed pregnancies and 1 in 6 unplanned pregnancies follow discontinuation for reasons other than the desire to become pregnant.^1^ This phenomenon of contraceptive discontinuation while women are in need poses a challenge and signals a deeper issue. Although family planning has long been a recognized right, if users are discontinuing a method while still in need, it may indicate that the right is not being assured by the family planning community of practice. Although some of these users will later decide to reinitiate use, others may never take up another method of contraception despite their desire to prevent pregnancy.^2^

An analysis of Demographic and Health Survey (DHS) data from 34 countries found that 38% of married women with unmet need were previously using a method of contraception.^3^ It has also been estimated that rates of unintended pregnancy would be 44%–81% lower if contraceptive failure or discontinuation while in need had not occurred.^1^ Discontinuation is especially common among young people who are known to discontinue methods at statistically higher rates than other users.^4^^,^^5^ Adolescents and unmarried women may face unique barriers and biases that can stand in the way of continued contraceptive use.

Person-centered, quality care is an essential component of the effective provision of health care services and systems; the United Nations Committee on Economic, Social, and Cultural Rights recognizes the right of access to available, accessible, and quality health care.^6^^,^^7^ This right to quality in care is accounted for in Bruce's and Jain's framework for family planning quality of care, a widely recognized framework in the family planning community.^8^^,^^9^ This framework outlines privacy, respect, confidentiality, and information exchange as integral components of a person's right to high quality when seeking care, particularly when interacting with a provider.^8^^,^^9^ Contraceptive counseling is a foundational moment in the client-provider interaction. During counseling, the 2 specific quality of care areas—as Bruce and Jain outlined—of interpersonal relations and information exchange are most evident. If a client clearly understands their options and has a better experience with their provider, it is suggested that they will feel better supported to choose a method that will enable a positive user experience and leave them more likely to desire continuing method use. Uncovering the links between improvements in quality of care in counseling and method discontinuation would allow the community of practice to better supply clients with counseling that addresses their needs, leaves them satisfied, and ensures their right to quality care.

Uncovering the links between improvements in quality of care in counseling and method discontinuation would allow the community of practice to better supply clients with counseling that addresses their needs, provides a positive experience, and ensures their right to quality care.

Evidence exploring predictors of discontinuation strongly suggests users' complex perspectives on, and experiences with, side effects is a factor that has the highest association with contraceptive discontinuation while in need. The World Health Organization (WHO) analyzed 60 DHS surveys from 25 countries and found that method-related concerns were the most common reason for discontinuation across all methods.^10^ Other analyses have found that the experience of side effects, as well as fears and misconceptions about the health consequences of irregular bleeding, play a leading role in contraceptive discontinuation and that adolescents have higher rates of discontinuation than older women.^11^^–^^14^ Addressing this common predictor of discontinuation during counseling could be pivotal to reducing unmet need and improving clients' satisfaction and user experience.

To better understand the current state of the evidence on the connections between counseling and the outcome of discontinuation, we undertook a scoping review of the literature available. This review sought to highlight what the evidence has documented on the relationship between client and provider information exchange and contraceptive discontinuation while in need. The primary aims were to summarize the global literature on contraceptive counseling approaches and to assess individual techniques and tools for counseling to identify elements of the client-provider interaction that may decrease rates of discontinuation while in need. A secondary aim was to assess the literature on contraceptive counseling approaches specific to the adolescent population, as they face greater barriers.

## METHODS

### Search Strategy

The scoping review focused on literature related to contraceptive counseling interventions and strategies and their outcome measurements to understand the link between counseling and outcomes such as contraceptive discontinuation. We searched 3 electronic databases, PubMed, EMBASE, and PsycINFO, using a broad approach with the following search terms: “contraception,” “contraceptive method(s),” “family planning,” and “contraceptive behavior,” “birth control”; together with “communication,” “education,” “conversation,” “consultation,” “counseling.”

We then repeated the above search strategy with the following additional terms: “intervention,” “strategy,” “outcome assessment,” “patient satisfaction,” “quality of care,” “quality of health care,” “decision making,” and “health behavior” to yield further literature.

To assess counseling priorities for youth, we used the above strategy and added the following terms: “youth(s),” “adolescent(s),” “teen(s),” “teenager(s),” and “young adult.” The concept of young adult (i.e., 24 years or younger) is relatively new, and many databases do not have this term until 2010 or after. As such, we conducted manual screening for this population.

Additionally, we identified unpublished papers in a gray literature search with Google Scholar and Google using manual search methods, using the above terms until only duplicate sources were identified, and no new sources were found.

### Inclusion Criteria

We only included publications in English. No literature was excluded based on country of origin. We included publications from January 1, 1990, to November 30, 2018. All study types and designs including qualitative, quantitative, and mixed methods, were considered; this includes systematic reviews, meta-analyses, commentaries, and technical reports.

### Study Selection

All resulting article titles and abstracts were screened by KL for inclusion. Peer-reviewed and gray literature were included if the publication described an intervention with a named outcome variable defining success or effectiveness of a contraceptive counseling-related intervention. Gray literature was limited to theses and technical reports. The reference list of identified publications was searched for additional sources. Commentaries were included if found to be substantive of the objectives of the review.

A cursory scan of the updated literature as of September 28, 2020, and snowball referencing was conducted by KD and yielded 9 additional articles for inclusion.

In total, the search resulted in 2,156 articles, of which 54 were ultimately included in the scoping review (Figure and Table). The most common reasons for initial exclusion included article type, including systematic reviews, letters to the editor, clinical commentaries, and perspectives publications. Seventy-four full-text articles were then assessed, and articles with no named outcome variable or intervention, irrelevant specialized focus (e.g., HIV patients accessing mobile health clinics), and commentaries were excluded.

**FIGURE:**
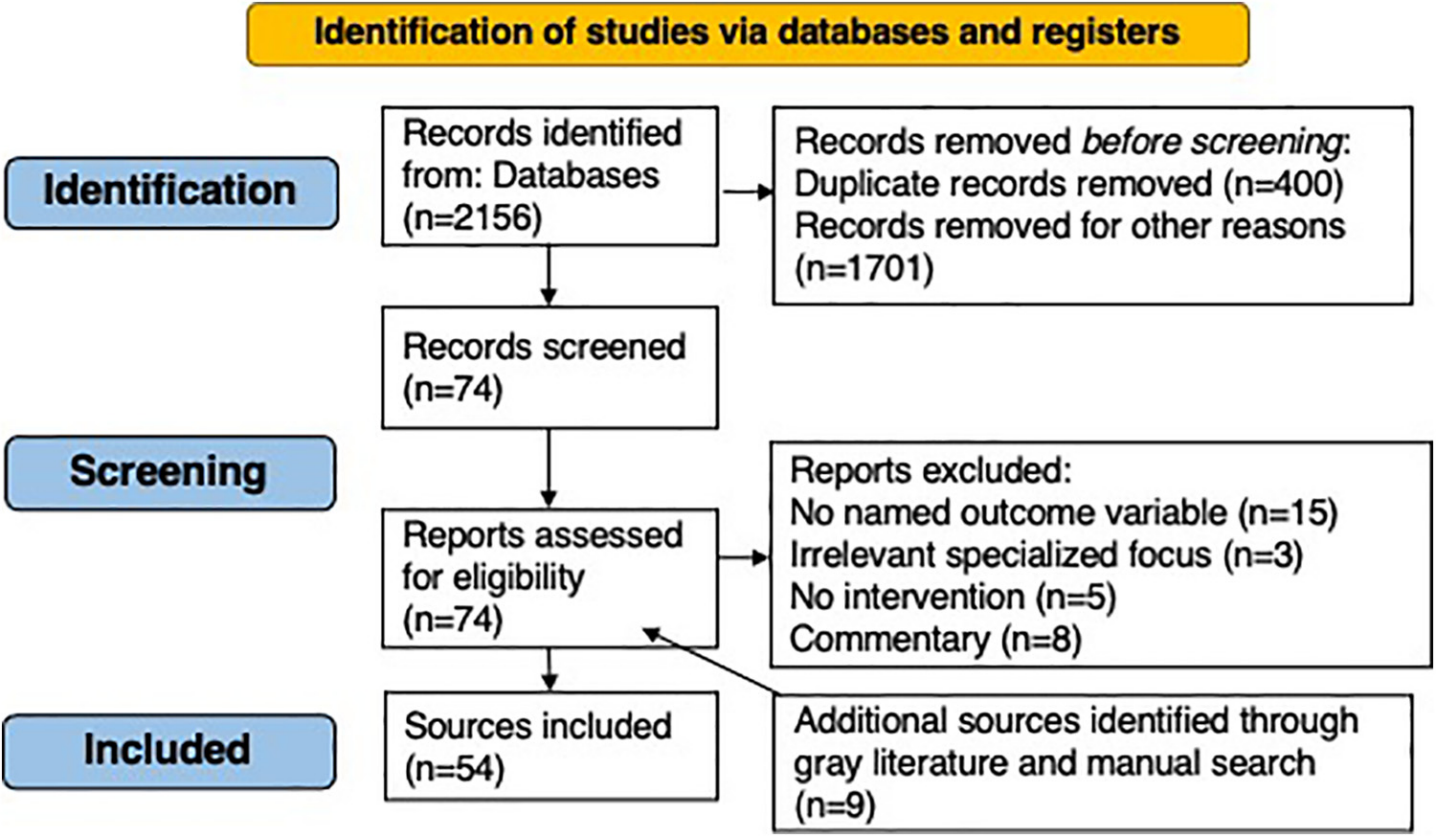
Flow Diagram of Search and Review Results of Evidence on Contraceptive Counseling Interventions and Strategies

**TABLE.** Details of Articles Included in Scoping Review of Client-Provider Interaction During Counseling and Contraceptive Discontinuation

Authors and Date	Key Finding Related to Contraceptive Discontinuation
Madden et al., 2013	U.S. study examined discontinuation seen in clients in the contraceptive CHOICE project; however, counseling was not a variable in the analysis. The authors did not set out to examine the effects of their contraceptive counseling approach and therefore did not look at its effects on discontinuation.
Liu et al., 2013	Higher quality of counseling, particularly measures of information provision, method choice, and interpersonal relations was associated with higher rates of continuation among DMPA-SC adopters in Nigeria.
Blanc et al., 2002	Analysis of DHS data from 15 countries showed a large proportion of discontinuation while in need caused by poor quality of the service environment, this discontinuation has a substantial effect on fertility outcomes. Mixed evidence on the use of discontinuation as an outcome indicator for the quality of care. More evidence is needed to understand the relationship between counseling and discontinuation.
Koenig et al., 1997	High quality of care was associated with a 72% higher likelihood of contraceptive discontinuation at up to 30 months in Bangladesh.
Sanogo et al., 2003	Quality of care in Senegal at the time of family planning adoption was a significant determinant of whether a client would be using contraception over 1.5 years later.
Kim et al., 1998	Analysis of counseling sessions in Kenya showed that contraceptive decision making and informed choice could be improved if providers took a more active role in counseling and related information provided to a client's circumstances and needs.
Fruhauf et al., 2018	From Burkina Faso, Ethiopia, Kenya, and Uganda, there was mixed evidence on the impact of quality (measured using a newly developed composite index) on contraceptive use. Discontinuation over time was not measured.
Chakraborty et al., 2019	Higher scores on the 3-question Method Information Index—measuring client-reported receipt of contraceptive information—were associated with continued use of family planning over 12 months among clients in Pakistan and Uganda.
Dehlendorf et al., 2016	U.S. study on quality of interpersonal care, particularly establishing rapport and eliciting the patient perspective measured using the Interpersonal Quality of Family Planning, influenced contraceptive use and continuation.
Dehlendorf et al., 2018	U.S. study on Interpersonal Quality of Family Planning showed positive associations with satisfaction with counseling and with the chosen method.
Abdel-Tawab and RamaRao, 2010	Inconsistent results showed observational evidence of a strong association between the client-provider interaction and continuation. However, evidence of interventions to improve counseling and impact continuation was not as strong.
Ramarao et al., 2003	Quality of care at the time of service delivery was positively associated with continuation at follow-up in the Philippines.
Abdel-Tawab and Roter, 2002	Analysis of counseling sessions in Egypt showed that client-centered counseling sessions, as opposed to provider-centered, were 3 times more likely to result in client satisfaction and method continuation at 7 months.
Jain et al., 2019	Quality of care among contraceptive adopters in India was predictive of method continuation in that clients who were provided counseling that scored higher on a 10-item index of quality that included information exchange and interpersonal relations were 3 times more likely to continue using their chosen method after 3 months.
Cotton et al., 1992	Women in Niger and the Gambia who reported they were not adequately counseled on side effects were more likely to discontinue their chosen method.
Nawar et al., 2004	After intervention to improve counseling in Egypt, including supportive supervision for providers, improving the facility's physical environment, and provider training, no effect was observed on discontinuation even though the client-provider interaction improved.
Leon et al., 2004	After an intervention to improve counseling in Peru, including provider training in the use of the Balanced Counseling Strategy, uptake among the intervention group improved but there was no observed effect on discontinuation.
Jain et al., 2012	Following an intervention to improve quality of care at the time of counseling in the Philippines, when the control and intervention groups were pooled, quality of care was found to be associated with discontinuation, but this effect was not seen when comparing between the control and intervention groups.
Modesto et al., 2014	Findings showed no significant differences between the intensive and routine counseling (on side effects) groups in Brazil on the discontinuation rates due to unpredictable menstrual bleeding of the 3 contraceptives. The authors concluded that routine counseling may be sufficient for many women to help reduce premature discontinuation rates and improve continuation rates and user satisfaction among new users of long-acting reversible contraceptive methods.
Lunde et al., 2017	Findings in the United States highlighted a need for better, anticipatory advising at the time of counseling that better prepared clients for side effects they may experience.
Villavicencio and Allen, 2016	Reviews the importance of supporting clients in the United States to understand and manage contraceptive-induced bleeding changes and highlighted the evidence supporting better anticipatory counseling as a strategy to improve rates of continuation.
Goldhammer et al., 2018	Women in Australia reported a desire for consistent and accurate contraceptive information and less bias from providers, regardless of age. Discontinuation was not evaluated.
Littlejohn and Kimport, 2017	Findings explored the different ways that providers in the United States discussed side effects during contraceptive counseling and highlighted the importance of counseling clients on the medical uncertainty of contraceptive side effect experience.
Canto de Cetina et al., 2001	Women in Mexico in the intervention group were provided with detailed pretreatment and ongoing counseling on common side effects of injectables and encouraged to return for follow-up visits, which was shown to lead to a higher likelihood of continuation.
Lei et al., 1996	Women in China in the intervention group were provided with intensive structured pretreatment and ongoing counseling on common side effects of injectables and encouraged to return for follow-up visits, which was shown to lead to a higher likelihood of continuation.
Grimes and Schulz, 2011	Authors asserted that counseling on side effects that was not optimistic may create a nocebo effect, whereby clients were more likely to report side effects.
Holt et al., 2018	Women in Mexico reported a desire for privacy, confidentiality, informed choice, and respectful treatment. They also wanted clear, complete, and correct information during counseling. The authors also highlighted variations in counseling preferences among groups of different ages and educational statuses. Discontinuation was not evaluated.
Teshome et al., 2017	In Ethiopia, among those counseled on family planning (n=139), women were significantly more likely to be satisfied with the family planning service they received if their provider discussed their partner's attitudes about family planning, and their own concerns about family planning. Discontinuation was not measured.
Donnelly et al., 2014	Findings demonstrated the different counseling priorities between clients and providers in the United States, in particular the elements of counseling that clients rank as most important—“how does the method work to prevent pregnancy,” and “is it safe”—vs. the providers' priorities of “how is it used” and “how often does a patient need to remember to use it.” No evaluation of discontinuation.
De la Vara-Salazar et al., 2018	Analysis of provider surveys in Mexico demonstrated variations in the quality of counseling between urban and rural providers, with rural providers providing better counseling overall. Cultural barriers to quality counseling are also discussed.
Brittain et al., 2018	Findings demonstrated that young people's preferences during counseling and highlights elements of counseling that are barriers to quality care. Young people value confidentiality, supportive client-provider interactions, specialized provider training, and the removal of logistical barriers to family planning.
Gomez and Wapman, 2017	Findings explored young U.S. Latinx and Black women's perceptions of their counseling experience and highlight the implicit pressure they receive and bias they perceive from their providers. Clients reported feeling pressured to choose a particular method, or family planning in general, and rapidly discontinuing these methods following these poor counseling experiences.
Johnson et al., 2010	Testing of the WHO decision-making tool in 3 countries showed that provider training in this tool resulted in better quality counseling overall, particularly regarding increased client participation, more tailored counseling, and better information exchange. Discontinuation was not evaluated.
Kim et al., 2005	Provider training in Mexico on the WHO decision-making tool resulted in better quality counseling overall, particularly about information exchange, more tailored counseling, and client involvement in decision making. Discontinuation was not evaluated in this study.
Chin-Quee et al., 2007	While clients in Nicaragua who were counseled by providers in the intervention group, those trained in the use of the WHO decision-making tool, reported an improved counseling experience, there was no significant difference between contraceptive use or discontinuation when compared to the control group.
Kim et al., 2003	Clients in Indonesia who were part of the intervention were coached on how to ask questions, express concerns, and seek clarifications. Participants in this group participated more fully in counseling sessions, asked more questions, and articulated concerns. There was a marginally significant effect on discontinuation, with participants in this group being less likely to discontinue use of their method after 8 months.
Whittaker et al., 2015	Study findings from the United States validated that the motivational interviewing technique could be effective with postabortion clients seeking family planning care.
Whittaker et al., 2016	Twice as many clients in the United States in the intervention group initiated a family planning method following motivation interviewing-based counseling. This group was more likely to still be using their method when followed up at 3 months.
Dehlendorf et al., 2019	After interaction with the My Birth Control app, clients in the United States in the intervention group rated their counseling session higher on measures of quality, but no effect was seen on contraceptive discontinuation.
Brandi and Fuentes, 2020	The authors argued that the use of tiered effectiveness as a primary aspect of counseling has the potential to undermine patient autonomy and choice.
Stanback et al., 2015	The authors advocated for the use of the tiered effectiveness tool to ensure clients are well informed about the effectiveness of their method options.
Marshall et al., 2017	This was an evaluation of U.S. client perceptions of the Birth Control Navigator but not a study of health outcomes or discontinuation.
Blitzer et al., 2017	This was not an evaluation of the Contraceptive Counseling and Care approach and did not explore discontinuation.
Holt et al., 2017	This was not an evaluation of the Quality in Contraceptive Counseling approach and did not explore discontinuation.
Dehlendorf et al., 2014	Review summarized best practices in counseling but did not directly address discontinuation. This was not an evaluation of these practices, but a review of the evidence for each best practice.
Hersh et al., 2018	The authors sought to validate the feasibility of using a GATHER-based counseling video instead of conversational counseling in Colombia. Discontinuation was not evaluated.
Rinehart et al., 1998	This was not an evaluation of the GATHER approach and did not explore discontinuation.
Callegari et al., 2017	The authors reviewed the evidence and discussed potential pitfalls of the Reproductive Life Planning approach and suggested alternatives. This was not an evaluation and did not explore discontinuation.
Nelson et al., 2016	Participants in this U.S. study often did not report well-defined reproductive health goals and the contraceptive methods they chose often did not align with their goals, so this highlighted the need for improved counseling.
Tyden et al., 2016	At follow-up, women in Sweden in the intervention group had better knowledge about reproduction compared to the control group, and they wished to have their last child earlier in life than at baseline. Client perspectives on the counseling they received were also overwhelmingly positive. Discontinuation was not evaluated.
Mittal et al., 2014	U.S. evaluation explored knowledge of contraception but did not explore contraceptive use and did not evaluate discontinuation.
Bommaraju et al., 2015	U.S. evaluation explored contraceptive use but did not evaluate discontinuation.
Rademacher et al., 2018	The NORMAL tool is a promising and evidence-based innovation for counseling on contraceptive-induced bleeding changes. No evaluations have yet assessed its effectiveness or impact on discontinuation.
Wyatt et al., 2014	Review identified the necessity for decision aids to be evidence-based, evaluated, and created in collaboration with intended users to guarantee relevant attributes are included (though they admit this can be challenging, as counseling priorities are different among groups of clients and over one's lifespan. The authors did not discuss discontinuation.

Abbreviations: DHS, Demographic and Health Survey; DMPA-SC, subcutaneous depot medroxyprogesterone acetate; GATHER, Greet, Ask, Tell, Help, Explain, Return;

NORMAL, Normal, Opportunities, Return, Methods, Absence of Menses, and Limit; U.S., United States; WHO, World Health Organization.

The 54 full-text articles were read and relevant data were extracted to describe: (1) the contraceptive counseling described in the article; (2) a description of any evaluation of the contraceptive counseling that occurred; and (3) the outcome measures (the Supplement includes additional details on articles).

## RESULTS

This scoping review first explores the evidence that broadly links the client-provider interaction during counseling with contraceptive discontinuation. Next, to better understand the intricacies of this relationship, we explore what clients and providers identify as their counseling priorities and preferences, with a specific focus on counseling around side effects. Lastly, we look at which individual approaches to counseling have been evaluated and the evidence of any impact that these interventions have on reducing, or not, contraceptive discontinuation.

For this review, authors consider contraceptive discontinuation while in need to describe users who voluntarily stop using their chosen method of contraception while they still desire to prevent pregnancy. As the methodologies and design of the research studies presented here may vary, the definitions of discontinuation used in these studies may also vary. The definitions used in each study can be found within the source documents, there may be some variation in whether authors consider discontinuation to encompass method switching. We have not attempted to describe these variations, nor did we exclude any research based on how discontinuation was defined.

### Quality Care, Client-Provider Interaction, and Discontinuation

#### Quality of Care and Contraceptive Discontinuation: Observational Evidence

While the umbrella term of quality of care is wide, common themes do emerge when reviewing researchers' explorations of the linkages between overall quality of care and discontinuation.

Blanc et al. assessed how discontinuation varies with the quality of the service delivery environment across 15 countries and whether discontinuation could be used as an indicator for the quality of services (measured by the Family Planning Program Effort score measuring the quality of services); the assessment relied on DHS analysis.^15^ They found that between 7%–27% of discontinuation for reasons other than a desire to become pregnant could be attributed to poor service delivery. Blanc et al. concluded that more research was urgently needed to understand the relationship between the counseling component of service delivery and discontinuation rates.^15^ This finding influenced subsequent family planning delivery interventions and motivated more emphasis on measuring and improving quality of care to prevent discontinuation among clients.^16^^–^^19^ Blanc et al. also conclude that the reduction of method failure and discontinuation rates can make a substantial contribution toward reducing unintended pregnancy.^15^ Several studies have explored which elements of the client-provider interaction may be related to method discontinuation. Chakraborty et. al highlight the important impact of information exchange on method discontinuation.^20^ Using data from a prospective cohort study in Pakistan and Uganda, the authors found that clients who had scored their counseling session higher on the Method Information Index—a measure of information exchange that looks at whether clients are informed about method options, potential side effects, and what to do if they experience side effects—were less likely to have discontinued their chosen method at 12 months.^20^

In 2016, Dehlendorf et al. conducted a large prospective cohort study in the United States that sought to determine if the quality of interpersonal care during contraceptive counseling was associated with contraceptive use over time.^21^ They found a positive effect on continuation at 3- and 6-months: patients who reported better interpersonal communication were more likely to continue using their chosen contraceptive method and to be using a highly or moderately effective method at 6 months. The most important factor noted by clients was “provider invests in beginning” and “eliciting the client's perspective,” which included greeting the client warmly, eliciting the client's concerns and preferences, using open-ended questions, making small talk, and showing interest in the impact of contraceptive use on the client's life.^22^

Abdel-Tawab and RamaRao assessed the association between client-provider interaction and contraceptive discontinuation through a scan of peer-reviewed publications and project reports.^23^ They found observational evidence of a strong positive association between the client-provider interaction and contraceptive continuation.^23^ Several observational studies have further detailed “good care” during the client-provider interaction—measured by provider responsiveness to client questions, appreciation of the need for privacy, provider trustworthiness and empathy, information provided, adequate time for consultation, and choice of methods provided—and found that it positively impacts contraceptive continuation.^16^^,^^17^^,^^23^^,^^24^ In their analysis of provider communication styles during counseling sessions, Abdel-Tawab and Roter showed that users who had been engaged in a consultation with a provider that measured well on a “client-centeredness index” with traits such as solidarity with the client, information giving, and facilitation—using language that encourages the client to express her ideas—were 3 times more likely to be continuing users at 3 months.^24^ Clients in this study were less likely to continue using their method if their provider used directive or negative language—such as statements of disagreement or “expressions of tension” such as “don't you believe me, I told you the IUD would be good for you.”^25^ In the most recent review discussed here, Jain et al. confirmed the predictive association between quality of care and method continuation.^26^ These authors evaluated the validity of a 10-item index measuring the quality of care and found that when clients were given information on the effective use of their selected method (including about side effects and how to use the method) and information on continuity of care (including information about the timing of their next visit and the possibility of switching to another method if their selected method was not suitable) they were 3 times more likely to continue use after 3 months.^26^

Liu et al. researched sociodemographic predictors of continued subcutaneous depot medroxyprogesterone acetate (DMPA-SC; brand name Sayana Press) injectable use after 3-months of initiation in Nigeria's private sector. As a part of the study, authors also sought to document counseling quality and side effect experiences as factors influencing discontinuation. Overall, Liu et al. found that higher quality of counseling, which was evaluated using measures of information provision, interpersonal relations, and method choice, was associated with higher rates of continuation. The authors suggested that even small differences in the quality of care provided at the time of method uptake can influence continuation. In the study, 70% of women reported that their provider asked or informed them of potential side effects; however, respondents rated the quality of this information exchange lower compared to other measures of quality.^14^ Other observational results echo these findings.^27^

While these observational studies demonstrate a clear association between the client-provider interaction and continuation, Abdel-Tawab and RamaRao also concluded that interventions to improve these interactions have not demonstrated a measured improvement in contraceptive discontinuation.^23^

Observational studies demonstrate a clear association between the client-provider interaction and continuation, but evidence shows that interventions to improve these interactions have not demonstrated a measured improvement in contraceptive discontinuation.

#### Quality of Care and Contraceptive Discontinuation: Interventions

Studies in Egypt and Peru, conducted by the Population Council, assessed the impact of different program interventions aiming to improve client-provider interactions on contraceptive counseling and subsequent method use.^28^^,^^29^ In Egypt, the intervention involved facilitative supervision for providers, improving the clinic's physical environment, and provider training focused on information provision and communication with clients about follow-up. Observed client-provider interaction improved, but there was no effect on discontinuation of the method, method change, or attainment of reproductive intentions, as measured at 6- and 13-months postintervention.^28^ In Peru, the intervention included provider training in counseling and the use of job aids, specifically, the Balanced Counseling Strategy. At the 1-year evaluation, the intervention resulted in more clients using contraception compared to the control group of clients who were not trained on discontinuation, but no observed effect on discontinuation.^29^ Other specific counseling interventions are described in more detail later in this article.

In 2012, Jain et al. published their report on a large longitudinal intervention study in the public sector of the Philippines evaluating the impact of provider training and implementation of a facilitative supervision program on the quality of care in family planning clinics where service providers were trained in effective information exchange.^30^ They found the intervention improved the provider's knowledge of the contraceptive technologies and quality of care reported by clients. The rate of unintended pregnancy decreased with improved quality of care within the intervention group—trending toward a statistically significant difference compared to the control group. When observed together, the likelihood of discontinuation did indeed decrease as the quality of care at the time of counseling improved; however, a comparison of the control and experimental groups did not show a significant effect of this provider-level training intervention on contraceptive use outcomes, including the likelihood of continuation, unintended pregnancy, or unwanted birth.^30^ Looking specifically at interventions focused on counseling on side effects, the findings are similarly mixed. For example, contrary to what the observational evidence would suggest, Modesto et al., in their randomized clinical trial, did not find any significant differences between the discontinuation rates of clients counseled intensively on side effects and bleeding disturbances when compared to a control group.^31^

Though there are promising interventions that improve the quality of care overall, these findings suggest that researchers have not, within this body of evidence, determined which elements of care create a client-provider experience that significantly impacts contraceptive discontinuation.

Promising interventions can improve the quality of care overall, but these findings suggest that researchers have not determined which elements of care create a client-provider experience that significantly impacts contraceptive discontinuation.

### The Importance of Counseling Around Side Effects

Side effects and the fear of side effects influence the client's decision to use and continue family planning methods, so it is important to consider how providers use the counseling session to prepare clients for what they may experience. We looked directly at the evidence landscape around how counseling practices can more effectively address side effects.

Each client's risk of experiencing side effects from their method of choice is unknown,^32^ especially the most cited reason for discontinuation, unscheduled bleeding.^33^ Yet, multiple studies emphasize that clients desire counseling on side effects that is tailored to their needs.^32^^,^^34^

Littlejohn and Kimport sought to examine how providers communicated this uncertainty to clients during contraceptive counseling through an observational study in the United States.^35^ Authors analyzed the audio transcripts of counseling sessions using qualitative methods. They found that providers presented the positive side effects (e.g., lighter periods) as side effects that are to be expected but presented the negative side effects (e.g., painful periods) as only possible. Serious side effects were not discussed in half the sessions. Authors posit that clients may discontinue a method if she finds that what she was counseled to expect does not match her experience.^35^

Further supporting this link between counseling on side effects and discontinuation, Canto de Cetina and Lei et al. found that women who received detailed pretreatment counseling and ongoing counseling on the common side effects of injectables—including the possibility of menstrual changes—and were encouraged to follow up with the provider, were more likely to continue using injectables than the control group.^36^^,^^37^

Though the evidence is strong that effective counseling on side effects is beneficial to the client, there are varying opinions on precisely how to best address side effects during counseling. Grimes and Schulz comment that by raising the potential for side effects (specific to oral contraceptives) with clients during a counseling session, providers could be inadvertently drawing their client's attention to the occurrence of these side effects, when otherwise the same client might not have noted the side effect or been aggravated by it.^38^ They encourage “optimistic counseling” that tells clients they will feel well. Since providers are aware that side effects are the most common reason for discontinuation of oral contraception, Grimes and Schulz argue that the power of suggestion might influence how they present information.^38^ While there is no rigorous evidence to support this hypothesis, it does suggest complex dynamics during the counseling experience that could impact discontinuation.

Overall, clients signal strongly that they desire thorough counseling on side effects, and there is some evidence to suggest that they will be less likely to continue using their chosen method of contraception if they are not properly counseled and prepared for possible side effects.

### Counseling Priorities and Preferences

Several studies have considered what clients themselves desire from their counseling sessions with a provider. These elements of the counseling experience are important to understanding how the experience could be improved to address contraceptive discontinuation.

Several studies looked at what clients desire from counseling to understand how to improve their counseling experience and address contraceptive discontinuation.

Holt et al. conducted a qualitative study with focus groups of women in Mexico to assess contraceptive counseling preferences.^39^ Women reported a desire for privacy, confidentiality, informed choice, and respectful treatment—key components of quality of care. Women desired clear, complete, and correct information and personalized counseling based on their needs, preferences, and prior method history, as opposed to counseling based on method effectiveness. Older, less educated women wanted the provider to give their own opinion about what method would be best for them, whereas younger women in the study, who typically had a higher education, desired more complete information about their options so that they could make an autonomous decision.^39^

Teshome et al. found in their descriptive cross-sectional study of 400 women attending prenatal clinics in Ethiopia that women were more likely to be highly satisfied with the counseling experience when asked about their partner's attitudes about contraception and their own concerns or worries about using family planning methods.^40^

Donnelly et al. used cross-sectional surveys to rank clients' and providers' priorities when receiving or sharing contraception information during counseling sessions.^41^ Women in this U.S.-based study rated, “How does the method work to prevent pregnancy?” as the most important consideration, with, “Is it safe?” as a close second. Providers rated, “How often does a patient need to remember to use it?” and “How is it used?” equally as the most important consideration. The greatest discrepancy between clients and providers in importance ratings was for religious acceptability, concealability, and method documentation for health insurance paperwork or medical notes, all of which were more important to providers.^41^ This study demonstrates how clients and providers may prioritize different information during contraceptive counseling and suggests that this may result in differing perceptions of the quality and effectiveness of the information exchange.

De la Vara-Salazar et al. found that cultural and societal factors also play an important role in the counseling experience.^42^ They conducted a country-wide cross-sectional study in Mexico to assess geographic and institutional factors associated with contraceptive counseling and to identify cultural barriers that providers perceive as limitations for their clients. The authors found that rural providers had better odds of offering adequate counseling, as determined by adherence to national standards and provider surveys. They speculated that this could be an effect of the close ties between providers and populations in rural areas of Mexico—leading to improved interpersonal communication during counseling. Additionally, authors documented that providers who perceived that their clients' religion would prevent them from asking about family planning services were less likely to provide satisfactory contraceptive counseling,^42^ revealing the importance of removing provider preconceptions from the counseling experience.

#### Counseling Priorities for Youth

While the literature on elements of counseling that are significant for youth is limited, the evidence available is important as contraceptive discontinuation among this age group is prevalent and contraceptive counseling during youth might be one of the first autonomous interactions clients have with health care providers. Brittain et al. conducted a systematic review of 37 articles and found that young people value confidentiality, supportive client-provider interactions, specialized provider training, and the removal of logistical barriers to family planning.^43^ Participants also cited refusal to provide certain methods, assuming the client needs contraception, rushed interactions, insufficient information about methods, and general demeanor that discourages the disclosure of sexual experiences as barriers to effective counseling.^43^

Gomez and Wapman conducted a qualitative study of contraceptive decision making among young Latinx and Black women aged 18–24 years in the United States. The authors found that the pressure these young women of color felt from their providers during contraceptive care, such as providers emphasizing some methods over others, tone of voice or affect, and minimization of or failure to adequately describe side effects, resulted in women adopting methods they quickly discontinued and impacted their intentions or ability to access contraceptive care in the future.^44^ Similar findings in Australia from Goldhammer et al. documented that young women aged 18–23 years wanted to be offered a full range of methods, regardless of their age (while also noting that they sensed provider bias due to their age).^34^ Women also reported they wanted very detailed counseling on potential side effects.^34^ Findings are echoed in a study by Lunde et al., where young women reported they wanted more concrete examples of possible side effects and personal narratives of side effects experienced by other young women.^32^ Young women wanted individual predictions of side effects—not non-concrete, general warnings. Further emphasizing the nuance and complexities of the counseling experience, users stated that although they were told about the possibility of side effects, they felt unprepared and did not expect the side effects they experienced. The authors identified the need for better, anticipatory advising at the time of counseling, including telling clients what to do if they experience side effects that the client finds unfavorable.^32^

Together, this evidence highlights that clients of all ages desire a relationship with their provider that exhibits respect and trust; they want correct and relevant information, especially about side effects; and they hope for a person-centered interaction that affords them the dignity of making an informed choice about their contraceptive use, free from provider bias.

Evidence shows that clients of all ages desire a relationship with their provider that exhibits respect and trust, correct and relevant information, and a person-centered interaction that affords them the dignity of making an informed choice about their contraceptive use, free from provider bias.

### Evidence for the Use of Specific Counseling Approaches to Impact Discontinuation

In summarizing the principles of good counseling, we wanted to better understand if and how existing counseling tools, techniques, and frameworks have integrated these priorities. We looked at whether current approaches to counseling had been evaluated for impact on family planning outcomes, including discontinuation.

While association between the overall client-provider interaction and discontinuation has been assessed,^15^ there has been less rigor in studying how using specific counseling approaches affects discontinuation. This review examined several distinct counseling practices and tools, of which just 5 have been directly evaluated for their impact on discontinuation: the WHO decision-making tool, Balanced Counseling Strategy, Smart Patient Coaching, Motivational Interviewing, and My Birth Control. The methodological design of the studies measuring the impact of these counseling approaches was varied.

The WHO Decision-Making Tool for Family Planning Clients and Providers, a job aid that uses a model of shared decision making between the provider and client, has been evaluated the most frequently, though outcomes of the evaluations are not consistent.^45^^–^^47^ The approach is a client-centered strategy that allows for technical expertise to be provided and for clients to express their needs, preferences, and concerns. This tool is a double-sided flipchart: 1 side is addressed to the client while a corresponding page is meant for the provider. Johnson et al. found that the tool improved provider's counseling performance (i.e., increased client engagement and more tailored counseling) and client communication (clients engaged in more decision making), but they did not measure contraceptive discontinuation.^45^ Other publications have evaluated this tool and found that it improved measures of information exchange both as a job aid to providers and as a decision aid to clients.^46^ However, Chin-Quee et al. was the only study to evaluate its impact on discontinuation and found that while using the tool demonstrated an improved counseling experience for the client, there was no demonstrable effect on discontinuation.^47^

The Balanced Counseling Strategy, a tool that aims to provide more tailored information to clients during counseling with the use of job aids, was evaluated by Leon et al.^29^ The study found that use of the tool did result in more clients using contraception. However, in this evaluation, there was no observed effect on discontinuation.^29^ Interestingly, while the Balanced Counseling Strategy is a commonly used counseling tool, the scoping review found no other evidence assessing its links with contraceptive discontinuation.

Smart Patient Coaching, an approach whereby an educator in the waiting room provides coaching to clients on how to ask questions, express concern, and seek clarifications from providers, was evaluated in Indonesia. Client's coached with this approach are reminded that “the provider wants to hear from them” and their subsequent interactions with providers were evaluated based on measures of how actively clients engaged in their counseling sessions. Smart Patient Coaching resulted in more shared decision making during counseling, with clients asking more direct and assertive questions after coaching. This had a small positive effect on contraceptive discontinuation at the 8-month follow-up (3.9% in the intervention group vs. 7.8% in the control group).^48^

Motivational interviewing is a patient-centered counseling style that seeks to create a collaborative relationship between the interviewer (provider) and client. It uses open-ended questioning, reflective listening, empathic statements, and exploration of ambivalence to obtain a client's intrinsic motivation for behavior change. Motivational interviewing trains providers to support clients to build confidence and self-efficacy. This approach was evaluated by Whitaker et al., who found that twice as many women (aged 15–29 years) in the motivational interviewing-based counseling intervention initiated and continued to use long-acting reversible contraceptives when followed up after 3 months compared to women who were not provided with those techniques.^49^^,^^50^

Most recently, My Birth Control—a tablet-based tool designed to help users select a contraceptive method that aligns with their values and preferences—was evaluated through a cluster-randomized design of family planning clients.^31^ The client is meant to interact with My Birth Control before visiting a provider, and clients in the control and intervention groups were followed up at 4 and 7 months. Clients in the intervention group rated their counseling higher on measures of quality, including informed decision and greater knowledge, but no significant effect was seen on contraceptive discontinuation at 7 months.^51^

The WHO has also developed the tiered effectiveness tool, a visual job aid that places contraceptive methods on a spectrum of how effective they are at preventing pregnancy with typical use. This tool is recommended by several professional organizations in the United States.^52^ Some in the family planning community, such as Stanback et al.,^53^ have advocated that informed choice can only be achieved if the client has information—especially about effectiveness—on all available methods. Others, however, have argued that this approach may undermine patient autonomy and choice.^52^ While this tool is commonly used during counseling and is often integrated into structured counseling approaches, no studies were found evaluating this tool for impact on discontinuation or other outcomes. There remains debate in the family planning community of practice over whether the effectiveness of a method should be the most prominent aspect of contraceptive counseling.

The scoping review identified other approaches, tools, and frameworks, such as The Birth Control Navigator (United States),^54^ Contraceptive Counseling and Care (Mexico),^55^ the Quality in Contraceptive Counseling Framework,^56^ Contraceptive Counseling Best Practices to Ensure Quality Communication and Enable Effective Contraceptive Use,^57^ GATHER (United States),^12^^,^^58^^,^^59^ reproductive life planning,^60^^–^^64^ and the NORMAL tool for counseling on contraceptive induced bleeding changes,^65^ but studies assessing use or the effectiveness of these different counseling approaches are limited, and none of them look at method discontinuation.

Finally, in an effort to pinpoint what attributes make for a good contraceptive counseling tool, Wyatt et al. assessed the qualities of the job aids themselves through a systematic review.^66^ Authors identify the necessity for decision aids to be evidence-based, evaluated, and created in collaboration with intended users to guarantee relevant attributes are included. But they admit this can be challenging, as counseling priorities differ among groups of clients and over one's lifespan. The authors did not discuss discontinuation.

While many approaches to counseling are commonly used, the evidence around their effectiveness is weak. When tools are evaluated, there is not enough evidence to validate their effectiveness when using contraceptive discontinuation as a primary indicator of effective and high-quality counseling.

When tools have been evaluated, contraceptive discontinuation is often not considered, leading to a lack of support to validate the tools' effectiveness.

## DISCUSSION AND IMPLICATIONS

For myriad reasons, users discontinue using contraception while they still desire to prevent pregnancy. The most prevalent reason seen here is method-related side effects and bleeding disturbances that are either intolerable for the user or lead to fears of infertility, pregnancy, or health issues. The practical implications of discontinuation can be severe: the client may face health risks—which may include unplanned pregnancy and unsafe abortion—if her need for contraception is not met. Since reasons for discontinuation while in need are complex, efforts to address it must consider all aspects of quality of care. This review finds that contraceptive counseling is an element of rights-based care that could be optimized to better support clients to choose methods that will enable a positive user experience; however, more research is needed to understand how the constellation of counseling strategies, techniques, and specific tools could be varied to reduce discontinuation while in need as an outcome.

Numerous observational studies reveal that the exchange of information between the client and the provider during counseling is associated with contraceptive discontinuation. In particular, the way that providers prepare clients for the side effects they might expect from their contraceptive options is of utmost importance. Clients should be invited to fully engage during their counseling session. They should be encouraged to participate in a dialogue to exchange information, ask questions themselves, seek clarifications, and share their concerns. In turn, providers should ensure that the information they offer about method options is relevant to the client's unique needs, providing them with the information they need to make an informed and voluntary choice about their preferred method.

In addition to the exchange of information, the relationship dynamics between the client and the provider have also been shown to have a measurable impact on the likelihood of method discontinuation. Ensuring that the client feels respected by the provider is an absolute priority. Trust and solidarity are key components of quality interpersonal relations. Clients are sensitive to explicit or implicit provider bias, and when perceived, this negatively impacts the client's counseling experience. Provider bias and preconceptions—around age, parity, religion, appropriate method choice, etc.—can lead to perceived pressure to choose a method that may not be right for the client, resulting in dissatisfaction with and discontinuation of her method. To avoid this, the provider should be trained in evidence-based approaches to recognize their own biases, elicit the client's perspective, listen actively, and be responsive to the client's needs. Indeed, provider bias may be a factor that is producing the mixed evidence around the impact of contraceptive counseling approaches to affect discontinuation. Without first addressing provider attitudes and perceptions, it is much more difficult to truly improve the quality of counseling all women receive.^67^

Provider bias may be a factor that is producing the mixed evidence around the impact of contraceptive counseling approaches on discontinuation.

When considering the subset of the literature reviewed that explores specific tools and practical approaches used during counseling, there is limited evidence to understand how these job aids and techniques impact contraceptive outcomes, such as discontinuation while in need. As counseling approaches evolve, it is important that they be optimized by integrating the elements of existing models that have, in this review, shown promise toward impacting discontinuation (e.g., techniques to improve the participation of the client in shared decision making, techniques and aids to support providers in counseling around side effects, and training for providers to ensure respectful, person-centered care, etc.). A summary of the principles of high-quality counseling that have been identified in this review can be seen in the Box. The community of practice should prioritize a better understanding of the association between these priorities and contraceptive discontinuation. If an evidenced-based understanding of this remains limited, family planning programs risk using tools and techniques that do not effectively lead to client satisfaction and voluntary continued use of their contraceptive method.

A scan of the literature presented here highlights regional variations in the breadth of evidence available. Many of the studies reviewed were implemented in mid- to high-income regions, like the United States and Europe. Indeed, when looking at the subset of literature exploring practical approaches to counseling, this review has not uncovered any research on the use of specific counseling tools or approaches implemented in sub-Saharan Africa, where access to methods is often limited and dissatisfied clients may face disproportionate barriers to follow-up care. This lack of evidence shows that we have not, as a research community, sought to understand how to address the specific counseling needs of these populations.

We must develop counseling approaches that respond to clients' needs and their individual (and often varied) priorities. This scoping review identified relatively few evaluations of widely available counseling tools. Although assessing the strength of the evidence of each is outside the scope of this review, it strongly suggests that we must thoroughly evaluate these approaches to understand the impact they might have on family planning outcomes, particularly discontinuation while in need.

BOX **Summary of Quality Counseling Principles**

- Counseling should be responsive to clients' individual priorities, questions, and concerns.
- Counseling should support shared decision making between the client and the provider, where the client's wishes come first. Clients should have the ability to choose which contraceptive method they want and should receive what they choose regardless of age, marital status, religion, or provider bias, etc.
- Counseling should ensure clients have confidence in the privacy and confidentiality of their sessions.
- Providers should display trustworthiness, empathy, and solidarity with the client, friendliness, and warmth. Encourage open-ended questions, dialogue, and listening.
- Counseling should encourage discussion around the client's reproductive health goals and their experiences and preferences for contraception. Discuss how individual methods can help them achieve those goals.
- Counseling should provide information that is accurate and relevant to the client, narrowing down the client's options based on their stated needs and preferences.
- Counseling should address and prepare clients for potential side effects of their method options and provide concrete examples of how these might affect their health and lifestyle.
- Counseling should prepare clients for follow-up, including the understanding of warning signs that require attention by a health care provider. Make a plan for follow-up appointments if necessary.

## CONCLUSION

Evidence suggests that there are links between the counseling clients receive from health care providers and their subsequent experience and behavior with their contraceptive method. However, the evidence on specific counseling interventions and approaches and their impact on contraceptive discontinuation is mixed and evidence gaps remain. Improving the quality of care through better interpersonal relations and improved information exchange during counseling is important. While discontinuation is not the only primary outcome of relevance, the impact of discontinuation on the incidence of unintended pregnancies is substantial. Although many common counseling approaches are currently used across contexts and programmatic interventions, there is limited evidence that existing tools and trainings improve the client-provider interaction and/or result in better health outcomes. When tools are evaluated, evidence is lacking to support that they are effective when thinking about discontinuation as an outcome.

It is also critical to remember that quality of care does not end at the moment a client has left a health facility with a method. Indeed, quality throughout the continuum of the care experience could be improved to address discontinuation. Effective counseling approaches that are grounded in the principles of person-centered quality of care should be rigorously studied within various contexts for their measured impact on health outcomes, especially contraceptive discontinuation while in need. These approaches and tools should then be prioritized for widespread dissemination and use.

## Supplementary Material

GHSP-D-21-00235-Supplement.pdf

## Notes

**Peer Reviewed**

**First published online:** November 9, 2021.
