# Supplementary material for: Leveraging the Client-Provider Interaction to Address Contraceptive Discontinuation: A Scoping Review of the Evidence That Links Them
Source: Glob Health Sci Pract. 2021 Dec 31;9(4):948–63. doi: 10.9745/GHSP-D-21-00235 (PMC8691884; doi:10.9745/GHSP-D-21-00235)
Supplement: GHSP-D-21-00235-Supplement.pdf [file GHSP-D-21-00235-Supplement.pdf]

Supplement to: Danna K, Angel A, Kuznicki J, et al. Leveraging the client-provider interaction to address contraceptive discontinuation: a scoping review of the evidence that links them. *Glob Health Sci Pract*. 2021;9(4). https://doi.org/10.9745/GHSP-D-21-00235

Supplement Table. Details of References Included in Scoping Review Results of Client-Provider Interaction During Counseling on Contraceptive Discontinuation

Title of reference	Authors	Year	Study design	Specific Counseling Approach or tool Evaluated	Country	Key finding related to discontinuation
Structured contraceptive counseling provided by the Contraceptive CHOICE Project	Madden et al.	2013	Longitudinal study examining method choice, uptake and subsequent contraceptive behavior among family planning clients	GATHER **(did not evaluate discontinuation)**	USA	Study examines discontinuation seen in clients in the contraceptive CHOICE project, however, counseling is not a variable in the analysis. Authors did not set out to examine the effects of their contraceptive counseling approach and therefore did not look at its effects on discontinuation.
Predictors of DMPA-SC continuation among urban Nigerian women: the influence of counseling quality and side effects	Liu et al.	2018	Longitudinal study of 541 DMPA-SC adopters	N/A	Nigeria	Higher quality of counseling, particularly measures of information provision, method choice, and interpersonal relations-was associated with higher rates of continuation.
Monitoring contraceptive continuation: links to fertility outcomes and quality of care	Blanc et al.	2002	Analysis of DHS data from 15 countries	N/A	15 countries	A large proportion of discontinuation while in need caused by poor quality of the service environment, this discontinuation has substantial effect on fertility outcomes. Mixed evidence re. the use of discontinuation as an outcome indicator for quality of care. More evidence needed to understand relationship between counseling and discontinuation
The influence of quality of care upon contraceptive use in rural Bangladesh	Koenig et al.	1997	Longitudinal, cross sectional household survey	N/A	Bangladesh	High quality of care associated with a 72% higher likelihood of contraceptive discontinuation at up to 30 months
Improving quality of care and use of contraceptives in Senegal	Sanogo et al.	2003	Longitudinal survey comparing quality of care and family planning outcomes following care at different facilities-some who had been trained in improved quality of care	N/A	Senegal	Quality of care at time of family planning adoption is a significant determinant of whether a client will be using contraception over 1.5 years later

Supplement to: Danna K, Angel A, Kuznicki J, et al. Leveraging the client-provider interaction to address contraceptive discontinuation: a scoping review of the evidence that links them. *Glob Health Sci Pract*. 2021;9(4). https://doi.org/10.9745/GHSP-D-21-00235

**Title of reference**	**Authors**	**Year**	**Study design**	**Specific Counseling Approach or tool Evaluated**	**Country**	**Key finding related to discontinuation**
Informed choice and decision-making in family planning counseling in Kenya	Kim et al.	1998	Analysis of transcripts from 176 counseling sessions	N/A	Kenya	Contraceptive decision making and informed choice could be improved if providers took a more active role in counseling and related information provided to a client's individual circumstances and needs
Measuring family planning quality and its link with contraceptive use in public facilities in Burkina Faso, Ethiopia, Kenya and Uganda.	Fruhauf et al.	2018	Evaluation of a composite measurement of quality of care using PMA2020 data	N/A	Burkina Faso, Ethiopia, Kenya, Uganda	Mixed evidence on the impact of quality (measured using a newly developed composite index) on contraceptive use. Discontinuation over time not measured.
Association Between the Quality of Contraceptive Counseling and Method Continuation: Findings From a Prospective Cohort Study in Social Franchise Clinics in Pakistan and Uganda	Chakraborty et al.	2019	Prospective cohort study of 1,998 family planning clients	N/A	Pakistan, Uganda	Higher scores on the 3-question Method Information Index (MII)—measuring client-reported receipt of contraceptive information—was associated with continued use of family planning over 12 months.
Association of the quality of interpersonal care during family planning counseling with contraceptive use	Dehlendorf et al.	2016	Prospective cohort study of 348 clients	N/A	USA	Quality of interpersonal care, particularly establishing rapport and eliciting the patient perspective measured using the Interpersonal Quality of Family Planning (IQFP), influences contraceptive use and continuation.
Development of a patient-reported measure of the interpersonal quality of family planning care	Dehlendorf et al.	2018	Cohort study of 346 women to test the validity of a patient report measure of quality of care	N/A	USA	IQFP showed positive associations with satisfaction with counseling and with the chosen method
Do improvements in client-provider interaction increase contraceptive continuation? Unraveling the puzzle	Abdel-Tawab, RamaRao	2010	Review of literature examining the impact of interventions to improve client-provider interaction	N/A	N/A	Inconsistent results show observational evidence of a strong association between the client -provider interaction and continuation. However, evidence of interventions to improve counseling and impact continuation is not as strong.

Supplement to: Danna K, Angel A, Kuznicki J, et al. Leveraging the client-provider interaction to address contraceptive discontinuation: a scoping review of the evidence that links them. *Glob Health Sci Pract*. 2021;9(4). https://doi.org/10.9745/GHSP-D-21-00235

**Title of reference**	**Authors**	**Year**	**Study design**	**Specific Counseling Approach or tool Evaluated**	**Country**	**Key finding related to discontinuation**
The link between quality of care and contraceptive use	Ramarao et al.	2003	Longitudinal study surveying 1728 new family planning users following service delivery and again 16+ months later.	N/A	Philippines	Quality of care at the time of service delivery is positively associated with continuation at follow up
The relevance of client-centered communication to family planning settings in developing countries: lessons from the Egyptian experience	Abdel-Tawab, Roter	2002	Analysis of audio transcripts from 112 client counseling sessions	N/A	Egypt	Counseling sessions that were client-centered, as opposed to provider centered were 3 times more likely to result in client satisfaction and method continuation at 7 months.
Validation of Two Quality of Care Measures: Results from a Longitudinal Study of Reversible Contraceptive Users in India	Jain et al.	2019	Longitudinal study of 2699 contraceptive adopters	N/A	India	Quality of care is predictive of method continuation in that clients who are provided counseling that scores higher on a 10-item index of quality that includes information exchange and interpersonal relations, they were three times more likely to continue using their chosen method after 3 months.
Early discontinuation of contraceptive use in Niger and the Gambia.	Cotton et al.	1992	Prospective cohort study of 650 new family planning clients in Niger and 570 in the Gambia examining contraceptive behavior	N/A	Niger, The Gambia	Women who reported they were not adequately counseled on side effects were more likely to discontinue their chosen method.
Impact of improved client–provider interaction on women's achievement of fertility goals in Egypt	Nawar et al.	2004	Longitudinal study comparing a control and intervention group (N=300 women each.)	N/A	Egypt	After intervention to improve counseling, including supportive supervision for providers, improving the facility's physical environment, and provider training, no effect was observed on discontinuation even though the client-provider interaction improved.
One-year client impacts of quality of care improvements achieved in Peru	Leon et al.	2004	Longitudinal study comparing a control (n=159 at baseline) and intervention group (n=179) up to 13 months after family planning counseling and adoption	Balanced Counseling Strategy **(Evaluated Discontinuation)**	Peru	After intervention to improve counseling, including provider training in the use of the Balanced Counseling Strategy, uptake among the intervention group improved but there was no observed effect on discontinuation.

Supplement to: Danna K, Angel A, Kuznicki J, et al. Leveraging the client-provider interaction to address contraceptive discontinuation: a scoping review of the evidence that links them. *Glob Health Sci Pract*. 2021;9(4). https://doi.org/10.9745/GHSP-D-21-00235

Title of reference	Authors	Year	Study design	Specific Counseling Approach or tool Evaluated	Country	Key finding related to discontinuation
Evaluation of an intervention to improve quality of care in family planning program in the Philippines	Jain et al.	2012	Longitudinal study evaluating the effect of improved quality of care interventions on the contraceptive use behavior and health outcomes of 1728 new family planning users	N/A	Philippines	Following an intervention to improve quality of care at the time of counseling, when the control and intervention groups are pooled, quality of care is found to be associated with discontinuation, but this effect is not seen when comparing between the control and intervention groups.
A randomized clinical trial of the effect of intensive versus non-intensive counselling on discontinuation rates due to bleeding disturbances of three long-acting reversible contraceptives.	Modesto et al.	2014	Randomized clinical trial with 297 women in a control or intervention group offered intensive counseling on bleeding changes and side effects	N/A	Brazil	Findings showed no significant differences between the intensive and routine counselling (on side effects) groups on the discontinuation rates due to unpredictable menstrual bleeding of the three contraceptives. Authors conclude that routine counselling may be sufficient for many women to help reduce premature discontinuation rates and improve continuation rates and user satisfaction among new users of LARC methods.
"Just Wear Dark Underpants Mainly": Learning from Adolescents' and Young Adults' Experiences with Early Discontinuation of the Contraceptive Implant	Lunde et al.	2017	Qualitative analysis of interviews with 16 young women presenting for removal of the contraceptive implant	N/A	USA	Findings highlight a need for better, anticipatory advising at the time of counseling that better prepares clients for side effects they may experience.
Unscheduled bleeding and contraceptive choice: increasing satisfaction and continuation rates.	Villavicencio, Allen	2016	Review outlining the potential bleeding irregularities with each method, as well as various treatment options, as a provider resource.	N/A	USA	Reviews the importance of supporting clients to understand and manage contraceptive induced bleeding changes and highlights the evidence supporting better anticipatory counseling as a strategy to improve rates of continuation.
What do young Australian women want	Goldhammer et al.	2018	Report on findings from the Contraceptive Use, Pregnancy Intentions and Decisions (CUPID), a	N/A	Australia	Women report a desire for consistent and accurate contraceptive information and less bias from providers, regardless of age.

Supplement to: Danna K, Angel A, Kuznicki J, et al. Leveraging the client-provider interaction to address contraceptive discontinuation: a scoping review of the evidence that links them. *Glob Health Sci Pract*. 2021;9(4). https://doi.org/10.9745/GHSP-D-21-00235

**Title of reference**	**Authors**	**Year**	**Study design**	**Specific Counseling Approach or tool Evaluated**	**Country**	**Key finding related to discontinuation**
(when talking to doctors about contraception)?			large-scale longitudinal study of 3,795 women			Discontinuation not evaluated.
Contesting and Differentially Constructing Uncertainty: Negotiations of Contraceptive Use in the Clinical Encounter	Littlejohn, Kimport	2017	Qualitative analysis of 102 contraceptive counseling visits	N/A	USA	Findings explore the different ways that providers discuss side effects during contraceptive counseling and highlight the importance of counseling clients on the medical uncertainty of contraceptive side effect experience
Effect of counseling to improve compliance in Mexican women receiving depot-medroxyprogesterone acetate	Canto De Cetina et al.	2001	Longitudinal case-control study among N=300 women exploring the effect of a counseling intervention on discontinuation	N/A	Mexico	Women in the intervention group were provided with detailed pretreatment and ongoing counseling on common side effects of injectables and encouraged to return for follow up visits, this was shown to lead to higher likelihood of continuation.
Effect of pretreatment counseling on discontinuation rates in Chinese women given depo-medroxyprogesterone acetate for contraception	Lei et al.	1996	Longitudinal case-control study among N=421 women exploring the effect of a counseling intervention on discontinuation	N/A	China	Women in the intervention group were provided with intensive structured pretreatment and ongoing counseling on common side effects of injectables and encouraged to return for follow up visits, this was shown to lead to higher likelihood of continuation.
Nonspecific side effects of oral contraceptives: nocebo or noise?	Grimes, Schulz	2011	Commentary on counseling around side effects	N/A	N/A	Author asserts that counseling on side effects that is not optimistic may create a nocebo affect, whereby clients are more likely to report side effects.
Women's preferences for contraceptive counseling in Mexico: Results from a focus group study	Holt et al.	2018	Qualitative analysis of 6 focus group discussions (N=43) with women	N/A	Mexico	Women reported a desire for privacy, confidentiality, informed choice and respectful treatment. They also wanted clear, complete and correct information during counseling. Authors also highlight variations in counseling preferences among groups of different ages and educational status.

Supplement to: Danna K, Angel A, Kuznicki J, et al. Leveraging the client-provider interaction to address contraceptive discontinuation: a scoping review of the evidence that links them. *Glob Health Sci Pract*. 2021;9(4). https://doi.org/10.9745/GHSP-D-21-00235

**Title of reference**	**Authors**	**Year**	**Study design**	**Specific Counseling Approach or tool Evaluated**	**Country**	**Key finding related to discontinuation**
						Discontinuation not evaluated.
Quality of family planning counseling among women attending prenatal care at a hospital in Addis Ababa	Teshome et al.	2017	Cross sectional study of 400 pregnant women	N/A	Ethiopia	Among those counseled on family planning (n=139), women were significantly more likely to be satisfied with the family planning service they received if their provider discussed their partners attitudes about family planning, and their own concerns about family planning. Discontinuation was not measured.
What matters most? The content and concordance of patients' and providers' information priorities for contraceptive decision making	Donnelly et al.	2014	Cross-sectional survey administered online to 417 women and 188 family planning providers	N/A	USA	Findings demonstrate the different counseling priorities between clients and providers, in particular the elements of counseling that clients rank as most important-- "how does the method work to prevent pregnancy", and "is it safe"—vs. the providers' priorities of "how is it used" and "how often does a patient need to remember to use it". No evaluation of discontinuation.
Providers perspective and geographic and institutional factors associated with family planning counseling	De la Vara-Salazar et al.	2018	Analysis of 16,829 cross-sectional surveys with family planning providers to identify geographic, institutional and cultural barriers for family planning clients	N/A	Mexico	Findings demonstrate variations in the quality of counseling between urban and rural providers, with rural providers providing better counseling overall. Cultural barriers to quality counseling are also discussed.
Youth-Friendly Family Planning Services for Young People: A Systematic Review Update	Brittain et al.	2018	Systematic review of literature	N/A	N/A	Findings demonstrate that young people's preferences during counseling and highlights elements of counseling that are barriers to quality care. Young people value confidentiality, supportive client-provider interactions, specialized provider training, and the removal of logistical barriers to family planning.

Supplement to: Danna K, Angel A, Kuznicki J, et al. Leveraging the client-provider interaction to address contraceptive discontinuation: a scoping review of the evidence that links them. *Glob Health Sci Pract*. 2021;9(4). https://doi.org/10.9745/GHSP-D-21-00235

**Title of reference**	**Authors**	**Year**	**Study design**	**Specific Counseling Approach or tool Evaluated**	**Country**	**Key finding related to discontinuation**
Under (implicit) pressure: young Black and Latina women's perceptions of contraceptive care	Gomez, Wapman	2017	Qualitative analysis of in-depth interviews from young women	N/A	USA	Findings explore young Latina and Black women's perceptions of their counseling experience and highlight the implicit pressure they receive and bias they perceive from their providers. Clients report feeling pressured to choose a particular method, or family planning in general, and rapidly discontinuing these methods following these poor counseling experiences.
Towards client-centered counseling: Development and testing of the WHO Decision-Making Tool	Johnson et al.	2010	Quantitative testing of a job aid developed to improve counseling with a total of 605 clients across 3 countries. Providers evaluated at baseline and again following training intervention.	The WHO Decision-Making tool **(No evaluation of discontinuation)**	Nicaragua, Mexico, Indonesia	Provider training in this tool resulted in better quality counseling overall, particularly with regard to increased client participation, more tailored counseling and better information exchange. Discontinuation not evaluated in this study.
Promoting informed choice: Evaluating a decision-making tool for family planning clients and providers in Mexico	Kim et al.	2005	Analysis of provider counseling sessions with clients at base line and following training intervention to improve counseling.	The WHO Decision-Making tool **(No evaluation of discontinuation)**	Mexico	Provider training in this tool resulted in better quality counseling overall, particularly with regard to information exchange, more tailored counseling and client involvement in decision making. Discontinuation not evaluated in this study.
Counseling tools alone do not improve method continuation: further evidence from the decision-making tool for family planning clients and providers in Nicaragua	Chin-Quee et al.	2007	Quasi experimental longitudinal surveys with family planning acceptors following counseling and service provision by providers in a control or intervention group	The WHO Decision-Making tool **(Evaluated discontinuation)**	Nicaragua	While clients counseled by providers in the intervention group, those trained in the use of the WHO decision making tool, did report an improved counseling experience there was no significant difference between contraceptive use or discontinuation when compared to the control group.
Increasing patient participation in reproductive health consultations: an evaluation of ‘‘Smart Patient’’ coaching in Indonesia	Kim et al.	2003	Analysis of sessions with 768 women, half of whom were part of an intervention to improve client participation during family planning counseling	Smart Patient Coaching **(Evaluated discontinuation)**	Indonesia	Patients who were part of the intervention were coached on how to ask questions, express concerns and seek clarifications. Participants in this group did, indeed, participate more fully in counseling sessions-asking

**Title of reference**	**Authors**	**Year**	**Study design**	**Specific Counseling Approach or tool Evaluated**	**Country**	**Key finding related to discontinuation**
						more questions and articulating concerns. There was a marginally significant effect on discontinuation, with participants in this group being less likely to discontinue use of their method after 8 months.
Motivational interviewing to improve postabortion contraceptive uptake by young women: development and feasibility of a counseling intervention	Whittaker et al.	2015	Analysis of client perspectives following abortion services to validate the feasibility of a novel counseling approach	Motivational Interviewing	USA	Study findings validated that the Motivational interviewing technique could be effective with post abortion clients seeking family planning care.
A motivational interviewing-based counseling intervention to increase postabortion uptake of contraception: A pilot randomized controlled trial.	Whittaker et al.	2016	Randomized controlled trial evaluating counseling sessions with providers through baseline and follow-up surveys of clients in a control group (n=31) and an intervention group (n=29) counseled by providers who were trained in Motivational Interviewing	Motivational Interviewing **(Evaluated Discontinuation)**	USA	Twice as many clients in the intervention group initiated a family planning method following MI-based counseling. This group was more likely to still be using their method when followed up at three months.
Cluster randomized trial of a patient-centered contraceptive decision support tool, My Birth Control	Dehlendorf et al.	2019	Randomized controlled trial analyzing post-visit surveys of 758 women to compare clients in a control group with those in the intervention who interacted with the My Birth Control tool.	My Birth Control **(Evaluated Discontinuation)**	USA	Following prior interaction with the My Birth Control app, clients in the intervention group rated their counseling session higher on measures of quality, but no effect was seen on contraceptive discontinuation
The history of tiered-effectiveness contraceptive counseling and the importance of patient-centered family planning care	Brandi, Fuentes	2020	Commentary on the use of the tiered effectiveness approach to counseling	WHO Tiered Effectiveness	N/A	Authors argue that the use of tiered effectiveness as a primary aspect of counseling has the potential to undermine patient autonomy and choice
WHO Tiered-Effectiveness Counseling Is Rights-Based Family Planning	Stanback et al.	2015	Commentary on the use of the tiered effectiveness approach to counseling	WHO Tiered Effectiveness	N/A	Authors advocate for the use of the tiered effectiveness tool to ensure clients are well informed about the effectiveness of their method options.

Supplement to: Danna K, Angel A, Kuznicki J, et al. Leveraging the client-provider interaction to address contraceptive discontinuation: a scoping review of the evidence that links them. *Glob Health Sci Pract*. 2021;9(4). https://doi.org/10.9745/GHSP-D-21-00235

**Title of reference**	**Authors**	**Year**	**Study design**	**Specific Counseling Approach or tool Evaluated**	**Country**	**Key finding related to discontinuation**
Patient perceptions of a decision support tool to assist with young women's contraceptive choice	Marshall et al.	2017	Qualitative interviews to understand client perceptions of the value of a (Decision Support Tool)	The Birth Control Navigator **(Discontinuation not evaluated)**	USA	This was an evaluation of client perceptions of this tool, but not a study of health outcomes or discontinuation
Contraceptive counselling and care: a personalized interactive approach	Blitzer et al.	2017	Paper summarizing the development of this approach	Contraceptive Counseling and Care (CCC) **(Discontinuation not evaluated)**	(Mexico)	This was not an evaluation of this approach and therefore did not explore discontinuation
Defining quality in contraceptive counseling to improve measurement of individuals' experiences and enable service delivery improvement	Holt et al.	2017	Commentary summarizing the development of the Quality in Contraceptive Counseling framework	Quality in Contraceptive Counseling	N/A	This was not an evaluation of this approach and therefore did not explore discontinuation
Contraceptive counseling: Best practices to ensure quality communication and enable effective contraceptive use	Dehlendorf et al.	2014	A review of the literature highlighting best practices	N/A	N/A	Authors summarize best practices in counseling but do not directly address discontinuation. This was not an evaluation of these practices, but a review of the evidence for each individual best practice.
Video compared to conversational contraceptive counseling during labor and maternity hospitalization in Colombia: A randomized trial	Hersh et al.	2018	Randomized controlled trial comparing clients counseled using a GATHER based video vs. those provided conversational counseling	GATHER **(did not evaluate discontinuation)**	Colombia	Authors sought to validate the feasibility of using a GATHER-based counseling video in lieu of conversational counseling. Discontinuation was not evaluated.
GATHER guide to counseling	Rinehart et al.	1998	Description of the GATHER approach	N/A	N/A	This was not an evaluation of this approach and therefore did not explore discontinuation
Addressing potential pitfalls of reproductive life planning with patient-centered counseling	Callegari et al.	2017	Commentary on the use of reproductive life planning during counseling	Reproductive Life Planning **(Discontinuation not evaluated)**	N/A	Authors review the evidence and discuss potential pitfalls of the Reproductive Life Planning approach and suggest alternatives. This is not an evaluation and there did not explore discontinuation.

Supplement to: Danna K, Angel A, Kuznicki J, et al. Leveraging the client-provider interaction to address contraceptive discontinuation: a scoping review of the evidence that links them. *Glob Health Sci Pract*. 2021;9(4). https://doi.org/10.9745/GHSP-D-21-00235

**Title of reference**	**Authors**	**Year**	**Study design**	**Specific Counseling Approach or tool Evaluated**	**Country**	**Key finding related to discontinuation**
Reproductive Life Planning and Preconception Care 2015: Attitudes of English-Speaking Family Planning Patients	Nelson et al.	2016	Survey of clients to explore the validity of the Reproductive Life Planning approach to counseling clients pre-conception	Reproductive Life Planning **(Discontinuation not evaluated)**	USA	Participants in this study often did not report well defined reproductive health goals and the contraceptive methods they chose often did not align with their goals, so highlighting the need for improved counseling
Using the Reproductive Life Plan in contraceptive counselling	Tyden et al.	2016	Analysis of 299 client perspectives on counseling comparing a control and intervention group (counseled using the reproductive life planning strategy)	Reproductive Life Planning **(Discontinuation not evaluated)**	Sweden	At follow-up, women in the intervention group had better knowledge about reproduction compared to the control group, and they wished to have their last child earlier in life than at baseline. Client perspectives on the counseling they received was also overwhelmingly positive. Discontinuation was not evaluated.
Use of a modified reproductive life plan to improve awareness of preconception health in women with chronic disease	Mittal et al.	2014	Pre and Post evaluation of 27 clients to assess their knowledge following counseling using the Reproductive Life Planning approach.	Reproductive Life Planning **(Discontinuation not evaluated)**	USA	Evaluation explores knowledge of contraception but not explore contraceptive use and does not evaluate discontinuation
Reproductive Life Plan Counseling and Effective Contraceptive Use among Urban Women Utilizing Title X Services.	Bommaraju et al.	2015	Analysis of data from 771 women receiving counseling from providers using the reproductive life plan approach, evaluation explores contraceptive use	Reproductive Life Planning **(Discontinuation not evaluated)**	USA	Evaluation explores contraceptive use but does not evaluate discontinuation
Menstrual Bleeding Changes Are NORMAL: Proposed Counseling Tool to Address Common Reasons for Non-Use and Discontinuation of Contraception	Rademacher et al.	2018	Discussion of the development and evidence behind the NORMAL tool for counseling on bleeding changes	NORMAL	N/A	The NORMAL tool is a promising and evidence-based innovation for counseling on contraceptive induced bleeding changes. No evaluations have yet assessed its effectiveness or impact on discontinuation.

Supplement to: Danna K, Angel A, Kuznicki J, et al. Leveraging the client-provider interaction to address contraceptive discontinuation: a scoping review of the evidence that links them. *Glob Health Sci Pract*. 2021;9(4). https://doi.org/10.9745/GHSP-D-21-00235

Title of reference	Authors	Year	Study design	Specific Counseling Approach or tool Evaluated	Country	Key finding related to discontinuation
Women's values in contraceptive choice: A systematic review of relevant attributes included in decision aids	Wyatt et al.	2014	Systematic review of evidence on various counseling job aids.	N/A	N/A	Authors identify the necessity for decision aids to be evidence-based, evaluated, and created in collaboration with intended users to guarantee relevant attributes are included (though they admit this can be challenging, as counseling priorities are different among groups of clients and over one's lifespan. Authors did not discuss discontinuation.
